# Comparison of machine learning models to predict the risk of breast cancer-related lymphedema among breast cancer survivors: a cross-sectional study in China

**DOI:** 10.3389/fonc.2024.1334082

**Published:** 2024-02-12

**Authors:** Jiali Du, Jing Yang, Qing Yang, Xin Zhang, Ling Yuan, Bing Fu

**Affiliations:** ^1^ Department of Breast Surgery, Sichuan Clinical Research Center for Cancer, Sichuan Cancer Hospital & Institute, Sichuan Cancer Center, Affiliated Cancer Hospital of University of Electronic Science and Technology of China, Chengdu, China; ^2^ Department of Nursing, Sichuan Clinical Research Center for Cancer, Sichuan Cancer Hospital & Institute, Sichuan Cancer Center, Affiliated Cancer Hospital of University of Electronic Science and Technology of China, Chengdu, China

**Keywords:** breast cancer, lymphoedema, machine learning, prediction model, cancer

## Abstract

**Objective:**

The aim of this study was to develop and validate a series of breast cancer-related lymphoedema risk prediction models using machine learning algorithms for early identification of high-risk individuals to reduce the incidence of postoperative breast cancer lymphoedema.

**Methods:**

This was a retrospective study conducted from January 2012 to July 2022 in a tertiary oncology hospital. Subsequent to the collection of clinical data, variables with predictive capacity for breast cancer-related lymphoedema (BCRL) were subjected to scrutiny utilizing the Least Absolute Shrinkage and Selection Operator (LASSO) technique. The entire dataset underwent a randomized partition into training and test subsets, adhering to a 7:3 distribution. Nine classification models were developed, and the model performance was evaluated based on accuracy, sensitivity, specificity, recall, precision, F-score, and area under curve (AUC) of the ROC curve. Ultimately, the selection of the optimal model hinged upon the AUC value. Grid search and 10-fold cross-validation was used to determine the best parameter setting for each algorithm.

**Results:**

A total of 670 patients were investigated, of which 469 were in the modeling group and 201 in the validation group. A total of 174 had BCRL (25.97%). The LASSO regression model screened for the 13 features most valuable in predicting BCRL. The range of each metric in the test set for the nine models was, in order: accuracy (0.75–0.84), sensitivity (0.50–0.79), specificity (0.79–0.93), recall (0.50–0.79), precision (0.51–0.70), F score (0.56–0.69), and AUC value (0.71–0.87). Overall, LR achieved the best performance in terms of accuracy (0.81), precision (0.60), sensitivity (0.79), specificity (0.82), recall (0.79), F-score (0.68), and AUC value (0.87) for predicting BCRL.

**Conclusion:**

The study established that the constructed logistic regression (LR) model exhibits a more favorable amalgamation of accuracy, sensitivity, specificity, recall, and AUC value. This configuration adeptly discerns patients who are at an elevated risk of BCRL. Consequently, this precise identification equips nurses with the means to undertake timely and tailored interventions, thus averting the onset of BCRL.

## Introduction

The most recent data derived from GLOBOCAN 2020 (1)showed that breast cancer, for the first time, has surpassed lung cancer as the most prevalent malignancy among women, with an estimated 2.3 million new cases globally, accounting for 11.7% of overall cancer incidence. Notably, advancements in early detection methods and therapeutic interventions have contributed to a notable enhancement in the 5-year relative survival rate for breast cancer patients. Over the span of the last three decades, this rate has surged from 79% to 91% (2).

As the survival prospects for breast cancer patients continue to improve, there arises an imperative to elevate the quality of life for women grappling with the complications of this ailment. Breast cancer-related lymphoedema (BCRL) emerges as a frequent chronic complication after breast cancer surgery. This condition typically arises due to the accumulation of protein-rich fluids within tissue interstitial spaces, occasioned by deficient lymphatic drainage (3). Lymphedema engenders swelling, distortion, and impaired functionality within the affected limbs, profoundly influencing both physical and psychological well-being, and the overall quality of life (4). However, investigations into the authentic prevalence of BCRL exhibit disparities, with reported incidence rates spanning from 6% to 83% (5). Despite recent trends indicating a decline in the occurrence of BCRL, the challenge of its treatment remains formidable. Effective management strategies for lymphoedema are still lacking, thereby underscoring the importance of a preventative approach to mitigate its onset.

A disease risk prediction model constitutes a statistical methodology rooted in the evaluation of various disease-associated risk factors. These factors are assigned scores in correspondence with their impact magnitude, and subsequently, the likelihood of an impending event is computed through a mathematical formulation (6). This model yields a more refined assessment of the likelihood of a particular outcome transpiring. This, in turn, facilitates the implementation of precisely tailored interventions targeting diverse risk cohorts, thus yielding a pronounced impact on augmenting patient prognosis (7).

The term “Machine Learning” (ML) was introduced by Arthur Samuel (8) in 1959. It denotes an assemblage of algorithmic techniques designed for the purpose of data representation and analysis, a domain that has found widespread application in the realm of cancer care research (9). ML methodologies demonstrate enhanced efficacy in addressing problems characterized by a profusion of potential predictors, in contrast to conventional statistical paradigms (10). While many studies within the domain of China have sought to devise predictive models for BCRL, a considerable subset of these inquiries have predominantly relied upon classical approaches such as logistic regression. These methodologies may deviate from ML algorithms with respect to key metrics such as accuracy, sensitivity, and specificity. It is pertinent to underscore that the arena of machine learning encompasses diverse algorithmic classifications, encompassing entities like support vector machines (SVM), decision trees (DT), and random forests (RF). The efficacy of predictive models forged through disparate algorithmic classifications inherently exhibits discrepancy. It is imperative to undertake intermodel comparisons for the problem of BCRL.

The objective of this study was to formulate and subsequently validate an array of predictive models with a specific focus on predicting the risk of BCRL, and to validate the model using data from a large sample retrospective cohort study. These models possess the potential to aid clinical practitioners in the identification of individuals who are predisposed to a heightened risk profile, which enable the timely implementation of targeted interventions, thereby contributing to a reduction in the occurrence of BCRL.

## Materials and methods

This was a cross-sectional study of consecutive cases diagnosed with breast cancer during the period 2021–2022. Ethical approval was obtained from Sichuan Cancer Hospital (Approval No. SCCHEC-02-2022-005).

### Study design

We conducted a survey-based cross-sectional study in a tertiary oncology hospital (Sichuan Cancer Hospital & Research Institute), the largest oncology hospital in Southwestern China, which guaranteed a sufficient sample size. Participants were recruited using the following criteria: (1) ≥18 years of age at the time of surgery; (2) patient diagnosed with breast cancer by histopathology and received surgical treatment for breast cancer; (3) patients with normal reading, comprehension, and expression skills; and (5) agreed to participate in the study. Exclusion criteria were as follows: (1) patients with other serious diseases, such as severe heart, cardiogenic, and renal failure; (2) surgical history and injury history of the affected upper limb before breast cancer surgery; (3) bilateral breast cancer; (4) lymphedema caused by other reasons, such as nephrogenic, cardiogenic, hypoproteinemia, deep vein thrombosis, and so on.

### Data collection procedures

The demographic, clinical, disease, treatment, and behavioral particulars of the patients were procured through the questionnaires and retrieval from the electronic medical record system (**Table 1**). The items of the lymphedema risk-reduction behavior checklist are shown in **Table 2**. Prior to the distribution of questionnaires, a comprehensive explanation regarding the study’s objectives and significance was conveyed to the patients and their families. Upon securing the patients’ consent, the individuals independently completed the demographic and behavioral data questionnaires. To ensure meticulous accuracy in information collection, a diverse array of methods was employed for data collection, including the retrieval of data from electronic medical records, utilization of the WeChat (a messaging application), and telephone interview.

**Table 1:** The study’s variable and their corresponding categories utilized in predicting BCRL.

Category	Variables
Demographics	Occupation, hypertension, diabetes, menstrual history, average monthly household income, mode of payment, residence, marital status, ethnicity, education level, weight, height, age
Clinical data	Tumor stage, pathological type, TNM stage of the tumor, axillary lymph node status, location of the tumor, hormone receptor status, HER-2 test status, Ki67 test status, recurrence of the tumor
Treatment types	Type of surgical incision, the number of lymph node positives, and the number of lymph nodes removed, level of axillary lymph node dissection (ALND), whether the affected side was the primary hand, endocrine therapy, adjuvant chemotherapy, postoperative radiotherapy, postoperative complications, lymph node surgical approach, breast surgical approach
Behavior-related information	The survey adhered to the “National Lymphedema Network (NLN)” as outlined by the NLN. These guidelines encompass a compilation of 18 preventive measures that are categorized into four distinct domains: mitigation of extreme temperature exposure, mitigation of compression on the upper extremities, lifestyle adjustments, and practices pertaining to skin care.

**Table 2:** Lymphedema risk-reduction behavior checklist.

No.	Item
Entry 1	Minor increases in edema of the upper extremities or chest should never be ignored, and edema of the upper extremities should be reported promptly.
Entry 2	No blood draws or injections in the affected limb and wearing lymphedema markers.
Entry 3	Avoid measuring blood pressure in the affected extremity; if bilateral upper extremity lymphedema is present, measure blood pressure in the lower extremity.
Entry 4	Keep the skin of the affected limbs clean and dry, pay attention to folds and finger gaps, and rub moisturizing lotion after bathing.
Entry 5	Avoid strenuous repetitive movements that increase resistance in the affected limb, such as scrubbing or pushing or pulling.
Entry 6	No lifting of excessively heavy objects (more than 5 pounds) and crossbody bags on the healthy side.
Entry 7	Do not wear necklaces or elastic bracelets that are too tight.
Entry 8	Avoid excessive temperature changes when drenching and washing dishes, avoid saunas or hot baths, and use sunscreen products.
Entry 9	Avoid injuries to the affected limbs, such as cuts, burns, sports injuries, insect bites and scratches.
Entry 10	Wear gloves when doing housework or planting flowers.
Entry 11	Avoid any injury when trimming nails.
Entry 12	Avoid excessive fatigue of the affected limb, rest and elevate the limb when it feels pain.
Entry 13	Wear elastic cuffs when flying with lymphedema patients, and use elastic bandages when flying long distances to increase fluid intake.
Entry 14	Wear light-weight breast implants or suitable bras without steel bra.
Entry 15	Shaving armpit hair with an electric shaver
Entry 16	Lymphedema patients are required to wear an elastic cuff during the day and are examined by the treating physician once every 4–6 months.
Entry 17	Report any signs of infection such as rash, itching, redness, pain, high skin temperature, or fever to your doctor.
Entry 18	Maintain an ideal body weight, eat a low-salt, high-protein, easy-to-digest diet, and avoid smoking and drinking alcohol.

### Evaluation of outcome

Diagnostic criteria for upper limb lymphedema: objective indexes were measured by circumferential measurement, and the circumferential diameters of the transverse carpal stripe, 10 cm above the transverse carpal stripe, elbow fossa, and 10 cm above the elbow fossa of the upper limbs of the bilateral upper limbs were measured with a non-elastic soft ruler. A difference of ≥2.0 cm between any point of the affected and healthy upper limbs was determined as breast cancer-related lymphedema. A difference of 6 cm was considered severe edema.

### Statistical analysis

Statistical analysis was performed using python (version: 3.7.0) and tableone package (version: 0.7.10) software. Because the data were obtained by standardized Z-scoring, they conformed to a normal distribution, where measures were described using mean ± standard deviation and counts were described using frequencies and percentages. An independent-samples T test was used to conduct the between-group comparisons. LASSO analysis was used to select the predictive variables. Before constructing the ML model, the input data are randomly divided into training and test sets. About 70% of the data is used for the training of the predictive model, and nearly 30% of the data is used for the validation of the test dataset. Recall, precision, accuracy, F-score, area under curve (AUC), sensitivity, and specificity based on the confusion matrix were used as the indicators for evaluating the predictive performance of the model.

## Results

### Patient participation and characteristics

On the basis of the circumferential measurements, 670 postoperative breast cancer patients were categorized into non-lymphedema and lymphedema groups, of which 496 (74.03%) were non-lymphedema patients and 174 (25.97%) were lymphedema patients. Comparisons between groups of the two groups of patients in terms of demographic, disease, and treatment information are shown in **Table 3**. The analytical outcomes underscored the presence of statistical significance across different categories of postoperative breast cancer patients in relation to attributes including BMI, hypertension, clinical staging, T-stage, N-stage, pathological type, age, type of surgery, type of lymph node surgery, surgical side, level of lymph node dissection, number of removed lymph nodes, number of positive lymph nodes, neoadjuvant chemotherapy, adjuvant chemotherapy, postoperative radiotherapy, endocrine therapy (P < 0.05).

**Table 3:** Comparison of demographic, disease, and treatment information of postoperative breast cancer patients between different groups.

Variable	Variable hierarchy	Total	Non-lymphedema	Lymphedema	P value
		(n=670)	(n=496)	(n=174)	
BMI, mean (SD)		24.1 (3.0)	23.8 (3.0)	25.1 (3.2)	<0.001
Hypertension, n (%)	No	561 (83.7)	425 (85.7)	136 (78.2)	0.028
	Yes	109 (16.3)	71 (14.3)	38 (21.8)	
Clinical staging, n (%)	0	26 (3.9)	25 (5.0)	1 (0.6)	<0.001
	I	132 (19.7)	122 (24.6)	10 (5.7)	
	II	354 (52.8)	257 (51.8)	97 (55.7)	
	III	115 (17.2)	63 (12.7)	52 (29.9)	
	IV	43 (6.4)	29 (5.8)	14 (8.0)	
Tumor stage, n (%)	T0	1 (0.1)	1 (0.2)		<0.001
	T1	207 (30.9)	177 (35.7)	30 (17.2)	
	T2	389 (58.1)	264 (53.2)	125 (71.8)	
	T3	28 (4.2)	17 (3.4)	11 (6.3)	
	T4	19 (2.8)	12 (2.4)	7 (4.0)	
	Tis	26 (3.9)	25 (5.0)	1 (0.6)	
N stage, n (%)	0	298 (44.5)	263 (53.0)	35 (20.1)	<0.001
	1	243 (36.3)	162 (32.7)	81 (46.6)	
	2	87 (13.0)	51 (10.3)	36 (20.7)	
	3	42 (6.3)	20 (4.0)	22 (12.6)	
Pathological type, n (%)	Invasive carcinoma	626 (93.4)	457 (92.1)	169 (97.1)	0.035
	Non-invasive carcinoma	44 (6.6)	39 (7.9)	5 (2.9)	
Age, mean (SD)		50.4 (10.0)	51.2 (9.7)	48.4 (10.6)	0.003
Type_of_surgery, n (%)	Mastectomy	509 (76.0)	357 (72.0)	152 (87.4)	<0.001
	Breast-conserving surgery	157 (23.4)	136 (27.4)	21 (12.1)	
	Breast reconstruction	4 (0.6)	3 (0.6)	1 (0.6)	
Type_of_lymph_node_surgery, n (%)	SLNB	248 (37.0)	231 (46.6)	17 (9.8)	<0.001
	ALND	422 (63.0)	265 (53.4)	157 (90.2)	
Surgical_side, n (%)	Non-dominant side surgery	336 (50.1)	266 (53.6)	70 (40.2)	0.003
	Dominant side surgery	334 (49.9)	230 (46.4)	104 (59.8)	
Level_of_lymph_node_dissection, n (%)	0	248 (37.0)	231 (46.6)	17 (9.8)	<0.001
	I level	11 (1.6)	8 (1.6)	3 (1.7)	
	I, II level	384 (57.3)	236 (47.6)	148 (85.1)	
	I, II, III levels	27 (4.0)	21 (4.2)	6 (3.4)	
Number_of_removed_lymph_nodes, mean (SD)		13.2 (8.1)	11.8 (7.9)	17.0 (7.8)	<0.001
Number_of positive_lymph_nodes, mean (SD)		1.8 (3.9)	1.2 (2.7)	3.3 (5.9)	<0.001
Neoadjuvant_chemotherapy, n (%)	No	415 (61.9)	322 (64.9)	93 (53.4)	<0.001
	Yes (with paclitaxel)	205 (30.6)	151 (30.4)	54 (31.0)	
	Yes (without paclitaxel)	50 (7.5)	23 (4.6)	27 (15.5)	
Adjuvant_chemotherapy, n (%)	No	180 (26.9)	151 (30.4)	29 (16.7)	<0.001
	Yes (with paclitaxel)	417 (62.2)	285 (57.5)	132 (75.9)	
	Yes (without paclitaxel)	73 (10.9)	60 (12.1)	13 (7.5)	
Postoperative_radiotherapy, n (%)	No	279 (41.6)	243 (49.0)	36 (20.7)	<0.001
	Yes	391 (58.4)	253 (51.0)	138 (79.3)	
Endocrine_therapy, n (%)	No	240 (35.8)	198 (39.9)	42 (24.1)	<0.001
	Yes	430 (64.2)	298 (60.1)	132 (75.9)	

**Figure 1:**
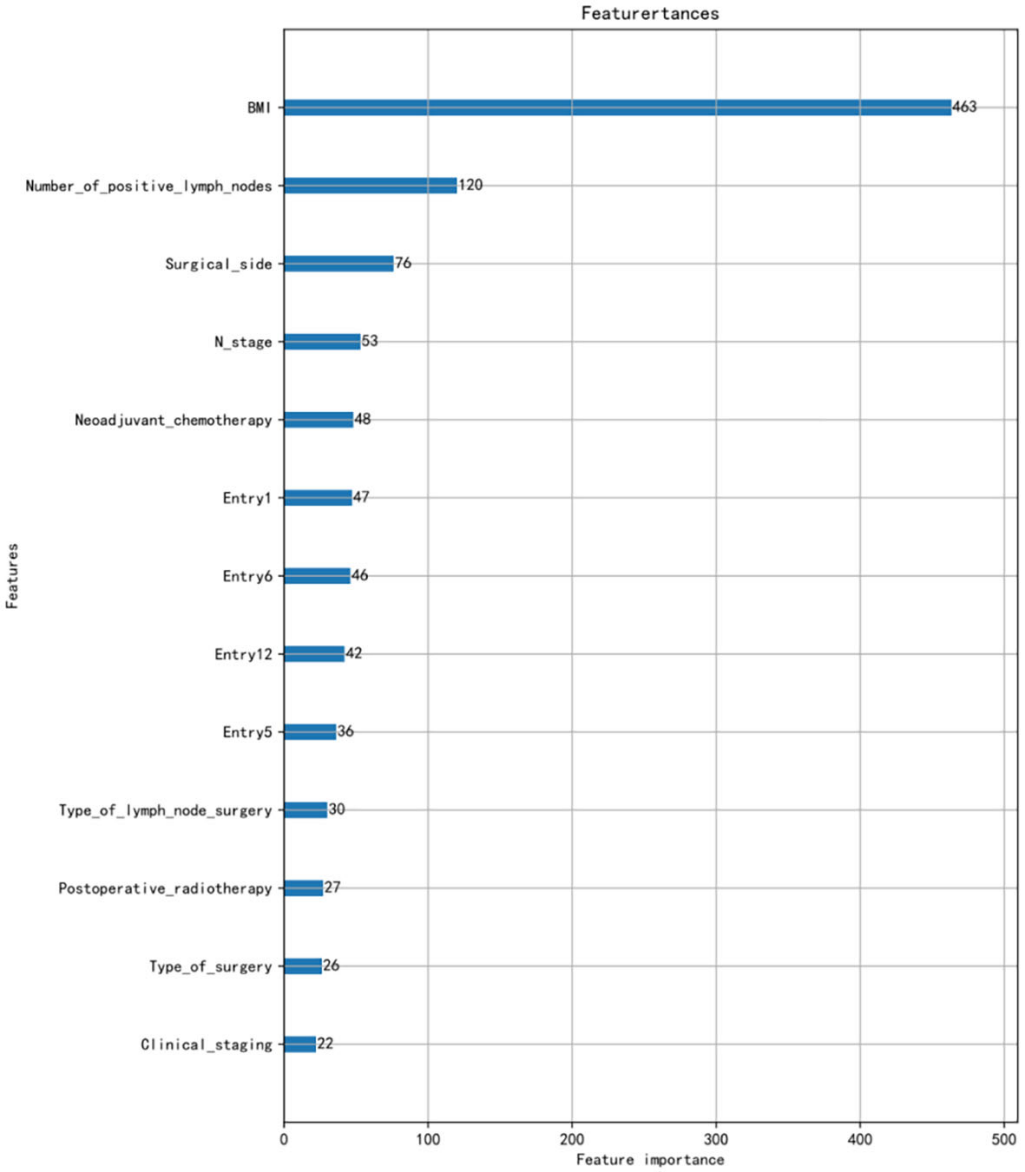
Importance ranking of variables.

Using LASSO regression for feature screening and cross-validation method to select the optimal model parameters, the 13 predictive values for BCRL were finally screened out. The 13 characteristics were as follows: BMI, number of positive lymph nodes, surgical side, N stage, neoadjuvant chemotherapy (NAC), type of lymph node surgery, postoperative radiotherapy, type of surgery, clinical staging, entry 1, entry 5, entry 6, and entry 12.The ranking of the importance of the 13 features screened for the results is shown in **Figure 1**.

### The ML models’ prediction performance

In this study, support vector machines (SVM), stochastic gradient descent (SGD), K-nearest neighbor (KNN), decision trees (DT), random forests (RF), extra trees (ET), extreme gradient boosting (XGBoost), light gradient boosting machine (LightGBM), and logistic regression (LR) were used for modeling in the training set and internally validated with the test set. This study used a method combining grid search and 10-fold cross-validation, which is realized by calling the GridSearchCV function in sklearn library. After finding the optimal parameter through GridSearchCV, it was directly brought into the model to observe the performance of the model.

**Figure 2:**
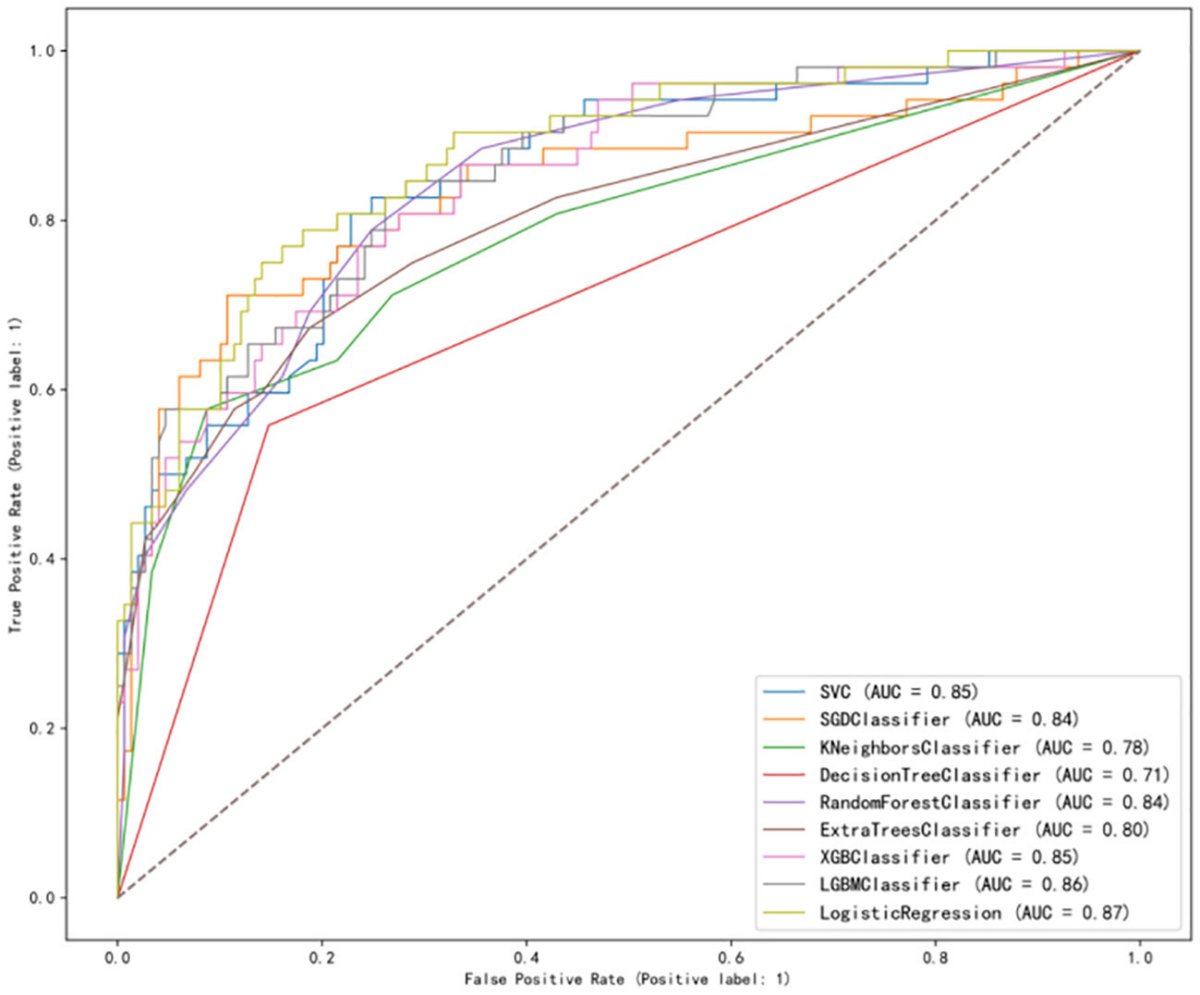
Receiver operating characteristic curve of the nine models in the validation set.

After analysis, the ROC curves for the nine models is shown in **Figure 2**. Internal validation and external validation were performed for each model category, followed by a comparison of the performance indicators of the nine models combined, and finally LR was found to have the best predictive performance based on the AUC values (0.87), with 81% accuracy, 82% specificity, and 79% recall for BCRL. The performance characteristics of the nine ML models for predicting BCRL are summarized in **Table 4**.

**Table 4:** Performance results for the machine learning models in the validation set.

Model	Accuracy	Sensitivity	Specificity	Recall	Precision	F-score	AUC
SVM	0.78	0.62	0.83	0.62	0.56	0.59	0.85
SGD	0.84	0.71	0.88	0.71	0.67	0.69	0.84
KNN	0.75	0.63	0.79	0.63	0.51	0.56	0.78
DT	0.78	0.56	0.85	0.56	0.57	0.56	0.71
RF	0.80	0.56	0.88	0.56	0.62	0.59	0.84
ET	0.82	0.50	0.93	0.50	0.70	0.58	0.80
XGBoost	0.82	0.58	0.91	0.58	0.68	0.62	0.85
LightGBM	0.82	0.60	0.89	0.60	0.66	0.63	0.86
LR	0.81	0.79	0.82	0.79	0.60	0.68	0.87

## Discussion

Our study found that lymphedema occurs in 25.97% of breast cancer patients, with consistent results in a review by Erickson et al. (11), which reported an overall incidence of upper extremity edema of 26%. However, the incidence of BCRL was 33.82%, 19.77%, and 6.8% in the studies by Xiao Xu (12), Xie Danping et al. (13), and Card et al. (14), respectively, which may be due to the variability of results due to the diversity of the study sites, follow-up times, diagnostic criteria, and measurement methods.

Since lymphedema is a chronic progressive disease, early detection or prevention is very important for both patients and healthcare professionals. By identifying risk factors for lymphedema, the likelihood of its occurrence can be predicted and appropriate strategies can be adopted to reduce these risk factors. In this study, 13 characteristics were screened by LASSO regression.

The most important risk factor was the patient’s BMI. In Ahmed and Dominick’s study of 1,287 and 2,431 breast cancer survivors (15, 16), BMI was also identified as a significant risk factor for lymphedema. In a study by Park et al. (17), BMI ≥25 kg/m^2^ was an independent risk factor for lymphedema in an analysis of 406 women who received preoperative neoadjuvant chemotherapy. Although considered one of the most important risk factors for BCRL, and despite the fact that accumulation of lymphocytes is known to increase adipocyte proliferation and differentiation (18), the mechanism by which elevated BMI increases the risk of secondary lymphedema is unclear. Green (19) suggested that a possible explanation for obesity-induced lymphedema is the inability of the lymphatic system of the affected limb to transport the amount of lymph produced, which can be demonstrated by abnormal lymphatic scintigraphy. Therefore, health education aimed at promoting dietary and lifestyle changes in breast cancer patients with a high BMI is important to help reduce the risk of postsurgical lymphoedema. This can be provided by specialized breast nurses as part of a holistic and individualized care plan. Although the primary impact of early dietary intervention on weight loss is beneficial in reducing the risk of lymphedema, potential secondary outcomes include a positive association with breast cancer recurrence, self-care, and overall health (20). Individuals with comorbidities such as diabetes, cardiovascular disease, or musculoskeletal disorders may benefit from lower BMI through dietary interventions or exercise, thereby improving quality of life.

The impact of lymph node status on the presence of LE is a topic of ongoing debate. In this study, the second most significant risk factor was the number of positive lymph nodes, aligning closely with the findings of Wu et al. (21). In the multivariate analysis conducted by Kwan et al. (22), the quantity of pathological lymph nodes demonstrated statistically significant prognostic value for the severity of lymphedema. A meta-analysis report suggests that an increased number of metastatic lymph nodes elevated the incidence of LE (23). A prospective survey on lymphedema involving 627 breast cancer patients who underwent mastectomy revealed that with each additional positive lymph node, the risk of developing BCRL increased by 1.091 times (24). Huang et al. (25) assert that the status of axillary lymph nodes significantly influenced the treatment and prognosis of breast cancer patients. This may be attributed to a higher number of positive lymph nodes leading to a broader scope of axillary lymph node dissection, resulting in prolonged radiation therapy and potential damage to axillary lymph nodes, upper limbs, and chest tissues. Conversely, a study comparing LE following SLNB and ALND in lymph node-negative and lymph node-positive breast cancer suggested that only ALND was associated with LE, regardless of lymph node status (26).

This study indicated that NAC was also a significant risk factor for the occurrence of BCRL. A prospective cohort study of 276 patients found that compared to preoperative surgery, patients undergoing ALND with NAC treatment had a twofold increased risk of BCRL development (27). Another study involving 409 ALND patients also discovered that NAC increased the risk of BCRL by 3.76 times and was independently associated with BCRL (28). A recent retrospective analysis of 596 breast cancer patients who underwent ALND and chemotherapy revealed that, compared to preoperative surgery, patients undergoing NAC with ALND had a 1.5-fold increased risk of BCRL (29). This finding was consistent with another retrospective study of 848 ALND patients, where NAC patients experienced more prolonged BCRL events compared to those receiving adjuvant chemotherapy (HR 1.39; 95% CI [1.05, 1.84]) (30). Similar associations were observed in populations other than those undergoing ALND. A retrospective analysis of 3,136 patients who underwent breast excision surgery reported a BCRL incidence of 10.4%, with NAC (HR 1.42; 95% CI [1.10, 1.84]) increasing the prevalence of BCRL (31).

The prevalence of most preventive behaviors is low due to postoperative health education and patient adherence to recommendations for preventive behaviors, such as sunburn, trauma, injections, and ipsilateral blood draws. Mosquito bites on infected limbs were the most common because they are not easily avoided; however, they usually have a minimal impact on the patient and few relevant studies have shown it to be a risk factor for BCRL. Air travel ranked second in prevalence. Air travel has received relatively little attention, hence its higher prevalence. Theoretically, air travel can have a deleterious effect on lymphedema. It is hypothesized that changes in cabin pressure during ascent and descent of the aircraft and the relatively low cabin pressure at high altitudes are responsible for this problem (32). However, there are conflicting indications in the published literature (33, 34). In the present study, among these behaviors, “do not ignore upper extremity edema,” “avoid strenuous exercise of the affected extremity,” “avoid heavy lifting of the affected extremity,” and “avoiding excessive fatigue of the affected limb” were associated with lymphedema. This may be due to overuse leading to excessive muscle tension in the affected limb, which disrupts the balance of lymphatic return and in turn induces lymphedema (35). Preventive behaviors associated with the risk of lymphedema remain controversial, and this uncertainty influences decision-making in very different ways for people at risk of lymphedema or for people with lymphedema. Further research is needed to determine whether they exacerbate lymphedema in postoperative breast cancer patients.

In addition, we performed a comprehensive comparison of the nine models. In terms of sensitivity, LR has the highest sensitivity, which means that the model has a relatively high identification rate for positive patients, and the specificity of LR is 0.82, which means that the identification of negative patients is generally correct, whereas SVM, SGD, KNN, DT, RF, ET, XGBoost, and LightGBM were all generally stable and had lower overall effectiveness scores than LR. In comparison with previous studies, Fu et al. (36) used five machine learning classification algorithms: decision tree with C4.5 and decision tree with C5.0, GBM, ANN, and SVM for predicting BCRL. Out of the five trained classifiers, the artificial neural network detected lymphedema with an accuracy of 93.75%, sensitivity of 95.65%, and specificity of 91.03%. The use of real-time symptom report that allows the use of web-and-mobile-based mHealth system in detecting lymphedema status is a strength of the study, which increases the predictive performance of the model compared with the LR model in this study. Notash et al. (37) used six classification algorithms including C5.0’s decision tree, KNN, SVM, LDA, Bayesian, and MLP to construct the BCRL prediction model, of which the SVM algorithm showed the highest sensitivity and was found to be the best model for predicting lymphedema, based on the accuracy obtained, the algorithm correctly detected the presence or absence of lymphedema in newly diagnosed patients in 88% of cases, which was slightly higher than the AUC of the LR in this study (0.87).Wei et al. (38) derived and evaluated six machine learning models, and the results showed that the LR model performed the best in the early detection of lymphedema with the best performance, AUC = 0.889 (0.840–0.938), sensitivity = 0.771, specificity = 0.883, accuracy = 0.825, and Brier score = 0.141, which is slightly lower than the sensitivity of LR (0.79) in this study. In summary, among the risk prediction models for upper limb lymphedema in postoperative breast cancer patients, the model constructed by the LR algorithm has a better predictive performance, which can guide clinical medical personnel to develop targeted BCRL prevention strategies.

## Conclusion

In this study, using LASSO regression modeling, the 13 features that were most valuable in predicting the occurrence of BCRL were screened and ranked in order of importance, and the results showed that BMI was the most critical factor among them. Then, the dataset was divided into training and test sets in the ratio of 7:3, and after comparing the performance indicators of the nine models combined, the LR model achieved the accuracy (0.81), precision (0.60), sensitivity (0.79), specificity (0.82), recall (0.79), F-score (0.68), and AUC value (0.87) of predicting BCRL with the optimal performance and was able to identify patients at high risk of BCRL more accurately. Overall, the findings emphasize the importance of implementing holistic and comprehensive care for patients with cancer, and nurses with expertise in lymphedema can play a key role in this regard.

## Data availability statement

The raw data supporting the conclusions of this article will be made available by the authors, without undue reservation.

## Ethics statement

The studies involving humans were approved by Sichuan Cancer Hospital Ethics Committee. The studies were conducted in accordance with the local legislation and institutional requirements. The participants provided their written informed consent to participate in this study.

## Author contributions

JD: Conceptualization, Data curation, Investigation, Methodology, Writing – original draft. JY: Data curation, Supervision, Writing – original draft, Writing – review & editing. QY: Software, Supervision, Writing – review & editing. XZ: Investigation, Software, Writing – original draft. LY: Methodology, Resources, Writing – original draft. BF: Data curation, Writing – review & editing.
